# Flavonoids from the genus *Polygonatum*: biological activities and biosynthesis mechanism

**DOI:** 10.3389/fnut.2025.1574182

**Published:** 2025-04-10

**Authors:** Xin-Pei Ye, Yan-Yu Hu, Yan-Xi Chen, Zhen-Xing Tang, Zhong-Bao Jiang, Yue Fu, Zi-Xin Wang, Er-Xu Pi, Gao-Feng Bian, Lu-E Shi

**Affiliations:** ^1^Department of Biotechnology, College of Life and Environmental Sciences, Hangzhou Normal University, Hangzhou, Zhejiang, China; ^2^School of Culinary Art, Tourism College of Zhejiang, Hangzhou, Zhejiang, China; ^3^College of Material, Chemistry and Chemical Engineering, Key Laboratory of Organosilicon Chemistry and Material of Education, Hangzhou Normal University, Hangzhou, Zhejiang, China

**Keywords:** *Polygonatum*, flavonoids, biological activity, biosynthesis pathway, active ingredient

## Abstract

The genus *Polygonatum* is a medicinal plant that has been used as food for a long time, containing various biologically active compounds, including polysaccharides, saponins, flavonoids, alkaloids, and many others. *Polygonatum*, like other Chinese herbal plants, can be employed as natural medicines, exhibiting a reduced incidence of adverse effects compared to synthetic pharmaceuticals. Flavonoids are key biomarkers that indicate the quality of the genus *Polygonatum*, and constitute one of the primary active ingredients. Additionally, flavonoids exhibit a range of nutritional, biological and health-promoting characteristics, including antibacterial, antioxidant, anti-inflammatory, antitumor, and hypoglycemic properties. This paper reviewed biosynthesis and bioactivities of flavonoids from the genus *Polygonatum*. We hope that this paper would ultimately serve as a valuable reference for the development of flavonoid-related functional foods from the genus *Polygonatum*.

## 1 Introduction

**Figure 1:**
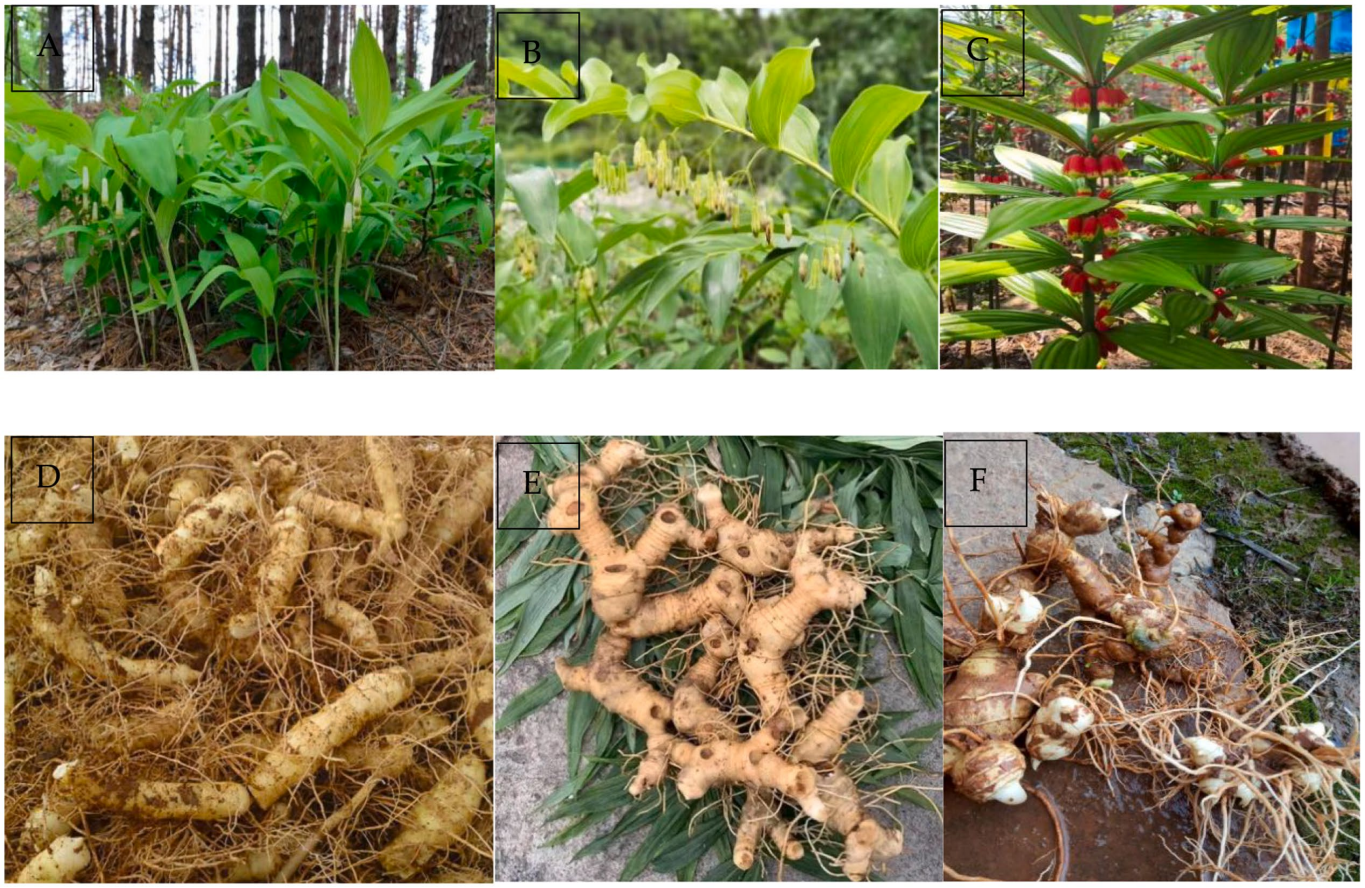
The plants of *P. odoratum* **(A)**, *P. cyrtonema* **(B)**, *P. kingianum* **(C)**; the rhizomes of *P. odoratum* **(D)**, *P. cyrtonema* **(E)**, *P. kingianum* **(F)**.

The genus *Polygonatum*, a perennial herb belonging to the Liliaceae family, is a distributed globally in the northern temperate zone, including in Russia, China, Japan, India, Korea, Europe, and North America. It comprises approximately 71 species and four varieties. In China, approximately 40 species in the genus *Polygonatum* are found, with the majority of these being cultivated the northeast of China, Hebei and other northern provinces (1–4). The majority of *Polygonatum* species are found in moist and shady environments, particularly in forested areas and fertile soil near bushes (4–6). Out of all the *Polygonatum* species, 37 species of them and one variety have been used for medicinal purposes, with the rhizome being the most commonly utilised part of the plant (7). In traditional Chinese medicine, *P. odoratum* rhizome is regarded as “Yuzhu,” while the rhizomes of *P. sibiricum*, *P. kingianum* and *P. cyrtonema* are classified as “Huangjing” (Figure 1), which have been incorporated into the Chinese Pharmacopoeia by 2020 (7, 8).

The genus *Polygonatum* has been used more than a history of 2,000 years of edible and medicinal use in China (9, 10). From ancient times to the present, the genus *Polygonatum* has been esteemed for its nutritional and medicinal properties. The genus *Polygonatum* is utilised in a variety of culinary preparations as part of a daily diet, including porridge, stews made with meat (chicken or pork), wine, and also as a vegetable (4, 8). In traditional Chinese medicine, the genus *Polygonatum* is regarded as a medicinal plant that possesses the ability to tonify the spleen and stomach, nourish the liver and kidneys, and thus is predominantly employed for the treatment of ailments, such as cough, dizziness, and lung trouble in clinical practice (11). Modern pharmacological studies have demonstrated that the genus *Polygonatum* exhibits a range of biological activities, such as antioxidant, anti-tumor, anti-inflammatory, anti-bacterial, anti-fatigue, hypoglycemic, and many others. These activities have been demonstrated to exert a certain therapeutic effect on a variety of diseases, including cardiovascular diseases, osteoporosis, cancers, gout, and diabetes mellitus (5, 6, 12–15). The diverse biological activities are ascribed to the active constituents present in the plant, including polysaccharides, polyphenols, lectins, flavonoids, alkaloids, and saponins, among others (6, 9). Given its high nutritional and medicinal values, the genus *Polygonatum* has significant potential for development and application in a range of fields, including clinical practice, health food, and cosmetics (16). Previously published work has reviewed the chemical compounds, bioactivities, and applications of the genus *Polygonatum* (4, 9, 17–20).

To date, the research on the genus *Polygonatum* has been primarily focused on the polysaccharides and saponins, with relatively limited investigation into flavonoids. However, in light of the recent expansion of the genus *Polygonatum* application fields, there has been a notable increase in interest in flavonoids within this genus. Flavonoids represent a significant class of secondary metabolites that are widely produced by the genus *Polygonatum*. These compounds play a pivotal role in growth and development of plants, and they demonstrate considerable potential for application in the fields of food and medicine (21–24). Flavonoids have been demonstrated to exert beneficial effects on human health, primarily as a result of their pronounced bioactivities, which include antibacterial, antioxidant, anti-inflammatory, anti-tumor, hypoglycemic properties, and many others (22, 23, 25, 26). The ingestion of flavonoids has been shown to reduce the risk of numerous chronic diseases. Moreover, the biosynthesis and bioactivities of flavonoids have been the focus of considerable research in the fields of plant biology and food for a considerable period (27, 28). Currently, the focus of research on flavonoids from the genus *Polygonatum* is on their extraction, bioactivities and biosynthesis pathway. Nevertheless, to the best of our knowledge, there is no review paper summarizing the biosynthetic processes and bioactivities of flavonoids from the genus *Polygonatum*. Accordingly, in present paper, we mainly reviewed biosynthetic pathways and biological activity of flavonoids derived from the genus *Polygonatum*. It would provide a theoretical foundation for the rational utilization of flavonoids in the genus *Polygonatum*, thereby facilitating the development of related products.

## 2 The biosynthesis pathway of flavonoids from the genus *Polygonaturm*

### 2.1 Flavonoids biosynthesis pathways

**Figure 2:**
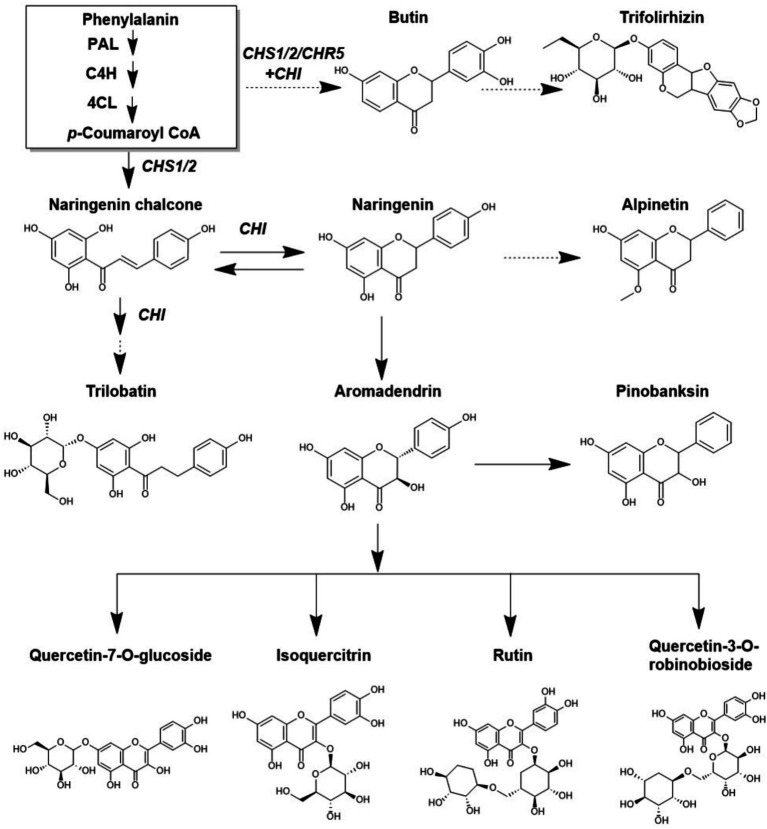
Metabolic flux of flavonoids pathway.

The metabolic pathway of flavonoids compounds has been widely studied in many plants. Flavonoids biosynthesis occurs at the junction of the shikimate and acetate pathways by providing *p*-coumaroyl and malonyl-CoA, respectively. Phenylalanine represents the initial product of the shikimate pathway. The synthesis of *p*-coumaroyl CoA is achieved through a series of catalytic reactions involving phenylalanine ammonialyase (PAL), cinnamic acid hydroxylase (C4H), and coumarin CoA ligase (4CL). Subsequently, various flavonoid compounds can be synthesized through the reaction of *p*-coumaroyl CoA and malonyl CoAs through chalcone synthase (CHS), chalcone reductase (CHR), and chalcone isomerase (CHI) (Figure 2).

### 2.2 Transcriptional regulation of flavonoids biosynthesis

The flavonoids biosynthesis is highly regulated by a complex network of biosynthetic enzymes and regulatory transcription factors (25–28). Numerous studies have identified and investigated the functions of various key enzyme-coding genes (e.g., PAL, C4H, 4CL, and CHS) within the flavonoids biosynthesis pathway (25–28). These functions have been thoroughly examined through *in vitro* assays of enzyme activity and transgenic assays. To date, the primary studies have focused on identifying the enzyme genes associated with flavonoids biosynthesis in the genus *Polygonatum* (29–31). Nevertheless, there are few research on the genes encoding a pivotal enzyme involved in flavonoids biosynthesis, which has hindered our understanding of the underlying mechanisms of this bioprocess in the genus *Polygonatum*. Zhang et al. (32) identified key enzyme genes associated with the isoflavonoid biosynthesis pathway through an analysis of *P. odoratum* transcriptome library, including seven key enzymes involved in isoflavonoid biosynthesis, namely, PAL, C4H, 4CL, CHS, CHI, 2-hydroxyisoflavone dehydratase, and isoflavone 7-*O*-glucosyltransferase. In a further comparison of amino acid sequences, the authors verified the expression of key genes for phenylalanine deaminase and CHI. Han et al. (33) also identified four novel characteristic genes (CHS1, CHI, CHS2 and CHR5) associated with flavonoids accumulation in *P. cyrtonema* using machine learning techniques. The functions of these genes affecting metabolic flux in the flavonoids pathway were validated by transient over-expression experiments in tobacco leaves. Recently, Chai et al. (34) employed transcriptome sequencing data from *P. sibiricum* to conduct a comprehensive analysis of the metabolic pathways associated with flavonoids. In particular, two key pathways were focused on flavonoids synthesis (ko00941), and flavonoids and flavonol synthesis (ko00944). In the flavonoids biosynthesis (ko00941) pathway, three genes encoded three key enzymes: trans-cinnamate 4-monooxygenase, CHS, and caffeoyl-CoA *O*-methyltransferase (EC:2.1.1.104). These enzymes facilitated the flavonoids biosynthesis. In contrast, the gene encoding CHI, cluster-2532.2507, has been demonstrated to inhibit the synthesis of compounds such as glycyrrhizin and naringenin. In the synthesis of flavonoids and flavonols (ko00944), only one up-regulated gene promoted flavonoid *O*-methyltransferase to catalyze the transfer of a methyl group to populin. The flavonoids biosynthesis pathway involves many genes, and their expression is highly correlated with the transcription level of these genes. Xiao et al. (35) employed transcriptome sequencing analysis to identify 10 differentially expressed genes associated with flavonoids biosynthesis in *P. kingianum*. qRT-PCR was employed to ascertain the expression quantities of the differential genes in different organs of *P. kingianum*. A correlation analysis was conducted to confirm the crucial role of CHS and CHI in the flavonoids biosynthesis pathway of *Polygonatum*, as evidenced by their correlation with flavonoids content. It was therefore concluded that the expression of these genes was responsible for the biosynthesis of flavonoids. Similarly, the work of Wang et al. (36) indicated that CHS and CHI played a crucial role in the flavonoids synthesis. Recently, Pan et al. (37) confirmed the role of CHS gene in the flavonoids synthesis in *P. cyrtonema*. In the transient overexpression of CHS, the expression level in the CHS group was found to be markedly higher than that in the control group, and the total flavonol content was also significantly higher, up to 1.83 times that of the control group.

It has been reported that some transcription factors families (e.g., MYB, bZIP, WD40, and NAC) are involved in regulating flavonoids biosynthesis in plants (23, 26–28). Plant MYBs are characterised by a highly conserved MYB DNA-binding domain and can be classified into four groups, such as 1R-MYB, 2R-MYB, 3R-MYB, and 4R-MYB. Xue et al. (38) found a correlation between the genes (MYB 3, MYB 97, MYB 102, MYB 33 and MYB 61) and the flavonoids content. This observation suggested that these genes might influence flavonoids production in *P. cytonema* through the regulation of the phenylpropanoid and anthocyanin pathways. In addition, bHLH transcription factors have been demonstrated to play pivotal roles in the regulation of flavonoids biosynthesis. In the investigation of Ye et al. (31), the authors found that among the transcription factors expressed in the rhizome of *P. cyrtonema* seedlings, bHLH was the main type, followed by ERF, MYB-related, C2H2 and NAC. Moreover, the differential expression of the genes associated with the flavonoids biosynthesis pathway in *P. cyrtonema* tubers was observed. As flavonoids concentration in the tubers increased, the authors successfully identified nine genes that were significantly up-regulated, three genes that were significantly down-regulated, and six genes that did not demonstrate significant alterations.

It has been demonstrated that plants experience a series of physiological and cellular changes when subjected to abiotic stresses, including UV, salt, low temperatures, heavy metal stress and drought stress, as well as biotic stresses, such as bacteria, fungi, nematodes, and insects (27, 28, 39–41). Flavonoids, which are secondary metabolites found in a variety of plants, have been shown to play an important role in handling a range of stresses. Various studies have indicated that genes associated with flavonoids biosynthesis (e.g., CHS, CHI, PAL, C4H, 4CL, NAC, MYB, bZIP, and bHLH) are regulated by abiotic and biotic stresses (42–44). The overexpression of these genes has been demonstrated to promote flavonoids biosynthesis and enhance plants tolerance to the stresses. However, the studies on the defensive role of flavonoids in the genus *Polygonatum* against various stresses are scarce. As with other abiotic stresses, antibiotic stress can also result in an elevation of flavonoids production in plants (42). In the study of Yang et al. (42), the findings indicated that both tetracycline hydrochloride (TCH) and sulfadiazine (SDZ) treatments resulted in the upregulation of the CSH gene. However, the TCH treatment exhibited a negligible impact on the CHI gene. The total flavonoids concentration was also found to be significantly higher in the SDZ treatment group compared to the TCH treatment group. As with many abiotic stresses, TCH and SDZ probably enhance the synthesis of flavonoids through the modulation of the expression of genes related to flavonoids and phenylpropanoid biosynthetic pathways, although to varying degrees.

## 3 The bioactivities of flavonoids from the genus *polygonatum*

**Figure 3:**
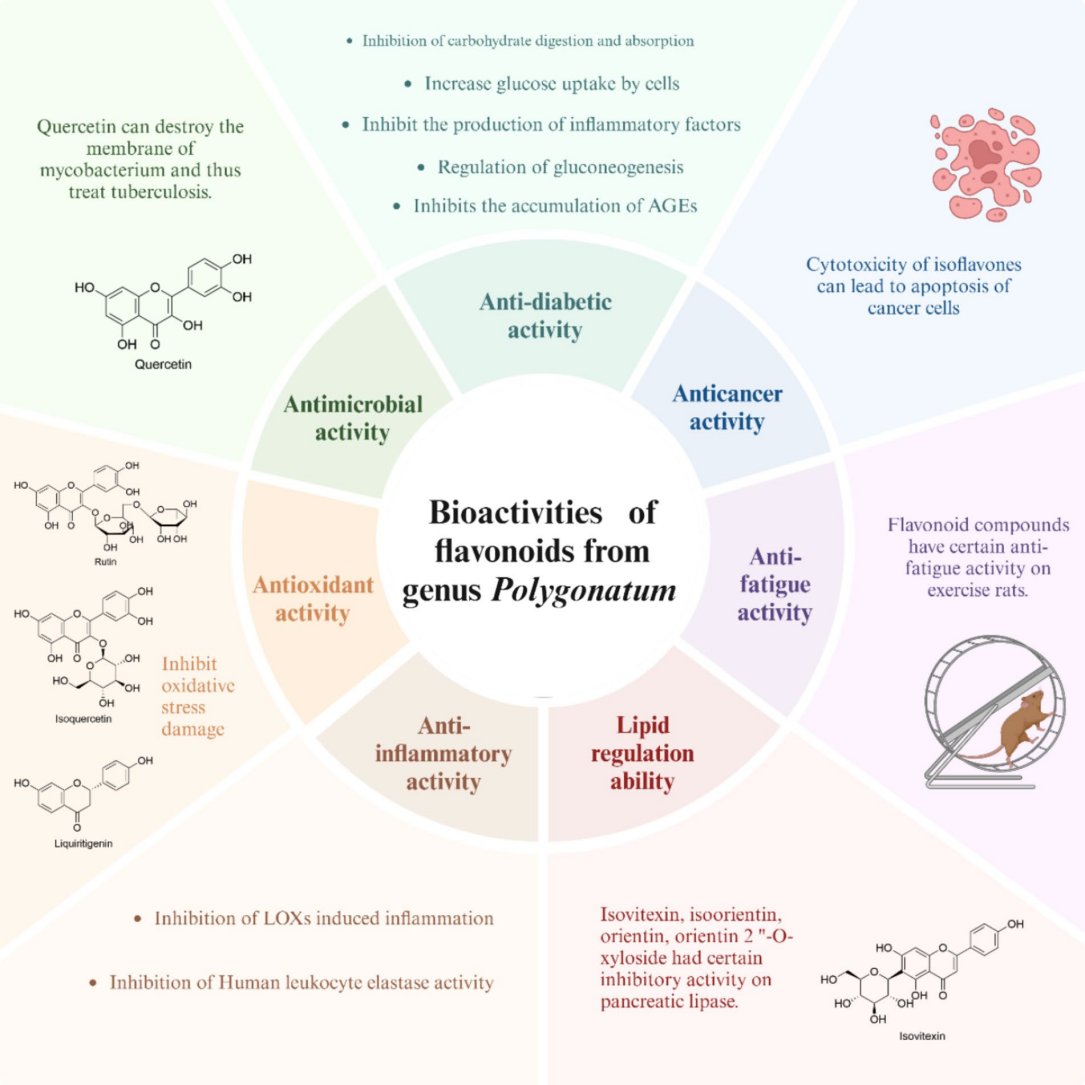
The biological activities of flavonoids from the genus *Polygonatum.*

As one of main constituents in the genus *Polygonatum*, flavonoids demonstrate a range of bioactivities, such as anti-diabetic, antioxidant, anti-fatigue, anticancer, and lipid-lowering effects, as illustrated in Figure 3. Among these activities, the researchers are particularly interested in the anti-diabetic and anti-oxidant properties of flavonoids.

### 3.1 Anti-diabetic activity

It is becoming increasingly clear that diabetes mellitus is having a significant impact on life quality of many individuals. The most effective drugs for treating diabetes mellitus are insulin and hypoglycemic agents, whereas these drugs have certain side effects on the human body. The genus *Polygonatum* has been demonstrated to possess beneficial properties with regard to the treatment and prevention of diabetes mellitus, owing to its secondary metabolites, namely the flavonoids. Recently, many anti-diabetic mechanisms of flavonoids derived from the genus *Polygonatum* have been reported (45, 46).

Digestive enzymes, including *α*-amylase and α-glucosidase, can metabolize dietary carbohydrates into glucose. This process occurs in the small intestine, where absorbed glucose elevates the postprandial blood glucose level. α-amylase and α-glucosidase inhibitors inhibit the digestion of dietary carbohydrates, consequently delaying glucose absorption and lowing postprandial blood glucose levels (10, 47, 48). Shu et al. (49) reported that total flavonoids of *P. odoratum* exhibited a favourable effect on regulating the levels of blood glucose. The authors determined the levels of blood glucose and insulin in streptozotocin (STZ)-induced type 1 diabetic mice and tetroxidine-induced type 2 diabetic mice after treatment with total flavonoids. There were no notable differences between the control group and the groups administrated with sugar or total flavonoids in STZ-induced type 1 diabetic mice, which exhibited complete *β*-cells damage to in the pancreatic islets. In contrast, pancreatic islet β-cells exhibited partial damage in rats with type 2 diabetes induced by tetracycline and a high-fat diet. Their insulin levels differed significantly from those of the control group, the gliclazide-treated group, and the total flavonoids-treated group. It is proposed that the total flavonoids could enhance the secretion of insulin secretion from pancreatic islet *β*-cells in mice with type 2 diabetes. The findings of *α*-amylase inhibition experiments provided further evidence that the total flavonoids remarkably suppressed the activity of *α*-amylase (49, 50). It can thus be postulated that the hypoglycaemic effect of the total flavonoids might be attributed to the suppression of α-amylase or the enhancement of glucose metabolism, and the increase of insulin secretion in pancreatic *β*-cells to improve insulin sensitivity. The mechanism may be analogous to that observed in the hypoglycaemic action of acarbose and gliclazide. Recently, Wang et al. (51) extracted the total flavonoids from *P. kingianum* and nine flavonoid components. IC_50_ values of the inhibition rates of *α*-amylase and α-glucosidase for purified and total flavonoids were 1.23 and 1.09 mg/mL, respectively. The findings indicated that the flavonoids from *P. kingianum* exhibited potential hypoglycaemic properties.

Another method of reducing blood glucose is to inhibit the intestinal absorption of glucose (52). The intestinal glucose absorption system is composed of two constituents: a mature constitute, sodium-dependent glucose transporter protein 1 (SGLT1) (49), which is a low-volume, high-affinity, active transporter protein; and a second constitute, glucose transporter protein 2 (GLUT2), which is a high-capacity, low-affinity, as well as readily translocatable protein that is associated with the majority of glucose transport through enterocytes (53, 54). Wang et al. (53) isolated three sappanin-type homoisoflavonoids derived from *P. odoratum* rhizomes, which exhibited strong inhibitory effects on GLUT2. The presence of sappanin-type homoisoflavones and flavonoid glycosides in *P. odoratum*, which might have a synergistic effect on lowing glucose levels in diabetic patients, and thus represented a promising basic ingredient for a new type of nutraceutical and functional food for glycaemic control.

Insulin resistance (IR) is an important pathogenic mechanism underlying the development of type 2 diabetes. It involves a reduction in the sensitivity of insulin target cells to a specific concentration of insulin, leading to impaired glucose uptake and utilization and a compensatory increase in insulin secretion (55). Zhang et al. (56) isolated nine homoisoflavonoids as well as isoflavone glycosides and flavonoid glycosides derived from *P. odoratum* rhizomes, and all eleven compounds demonstrated insulin-sensitising effects on 3 T3-L1 adipocytes using glucose uptake assays, showing that flavonoids might possess a potential for use as insulin sensitizers. Chen et al. (57) found that isorhamnetin, a flavonoid present in *Polygonatum*, exhibited overlap with IR disease targets, which were mainly involved signalling pathways such as PI3K-Akt, mTOR, and VEGF through network pharmacological analysis. Furthermore, cellular experiments demonstrated that isorhamnetin significantly enhanced glucose consumption in the IR model of HepG2 cells, increased the expression levels of PI3K and AKT1 proteins, and decreased the expression levels of VEGF and mTOR proteins. It was proposed that the combination of the active constituents of *Polygonatum*, such as flavonoids, and the key targets might be a crucial factor in *Polygonatum*’s efficacy in treating IR. Similarly, Zhang et al. (120) studied the mechanism of improving IR by bioactive components in *Polygonatum* through network pharmacology. The results showed that baicalein, sterols, flavonoids and other active components could improve IR by acting on the postsynaptic membrane, changing cell sensitivity to the drugs, cell neurotransmitter receptor activity and other biological processes, and regulating calcium ions, hormone resistance and other signalling pathways.

Adenosine monophosphate-activated protein kinase (AMPK) is a key sensor of cellular energy and a primary regulator of metabolic balance. It’s a crucial factor in understanding of diabetes and related metabolic disorders. Guo et al. (58) isolated six homoisoflavonoids, and one dihydrochalcone derived from *P. odoratum* rhizomes. Among the isolated compounds, homoisoflavonoids and dihydrochalcone were observed to significantly promote the phosphorylation of AMPK and acetyl coenzyme a carboxylase. These four compounds exhibited structural similarities in their *α*- and *β*-rings, while homoisoflavonoids differed only in their C-8 substitution patterns. Although compounds contained 3-hydroxy groups, their activities were significantly lower than those of compounds without 3-hydroxy groups. This suggested that the group of C-3 methyl exerted a crucial influence on the activity. Homoisoflavonoids and dihydrochalcone have been demonstrated to activate AMPK, and thus represent a promising class of compounds for the treatment of diabetes.

Advanced glycation endproducts (AGEs) are irreversible endproducts of protein glycosylation in the body, which massively accumulate in the body or in the tissues associated with major diseases such as diabetes, thereby causing an overload of the body’s glucose metabolism and further accelerating the formation of AGEs. AGEs have been linked to hyperglycaemia and the development of long term complications associated with the diabetes, including nephropathy, neuropathy and retinopathy (59). Dong et al. (60) obtained the ethanolic extract of *P. prattii* and identified three homoisoflavanones as active components using a STZ-induced diabetic rat renal AGEs accumulation model and an *in vitro* bovine serum albumin-glucose assay. The findings suggested that these compounds might have the potential to be utilised as a natural medicine for treating diabetic complications. Zhu (61) also isolated three homoisoflavanones from *P. odoratum*, which exhibited protective capacity in STZ-induced diabetic mice. The findings demonstrated that the therapeutic mechanism of homoisoflavanones for treating the diabetes might be due to the inhibition of AGEs formation.

Peroxisome proliferator-activated receptors (PPARs) have been demonstrated to play an important role in regulating lipid metabolism and glucose homeostasis. Furthermore, the use of PPARγ agonists has been shown to effectively improve IR in diabetic patients. Lin (62) determined the anti-PPARs ability of homoisoflavonoids isolated from *P. odoratum*. The findings revealed that both homoisoflavonoids activated PPARγ-dependent promoters, such as SHP, PPRE (PPARγ response element), and ABCA1 gene promoters, through a dose-dependent manner. It was indicated that these homoisoflavonoids possessed the ability to act as PPARγ agonists.

### 3.2 Antimicrobial activity

Many investigations have been carried out to investigate the antimicrobial mechanisms of flavonoids, which include the disruption of cell membranes and cell walls, the alteration of energy metabolism pathways, and the inhibition of biomolecule synthesis. Flavonoids, such as naringenin and quercetin, have been demonstrated to have the ability to disrupt the mycobacterial membrane of *Mycobacterium*, thereby providing a potential avenue for the treatment of tuberculosis. Additionally, the studies have showed that flavonoids, homoisoflavonoids, flavonols, and flavanones exhibited robust antifungal and antimicrobial properties (63, 64). Khan et al. (65) investigated that the crude extracts of *P. verticillatum* rhizome exhibited bacteriostatic activity. The findings indicated that the extracts exhibited antibacterial activity against *Escherichia coli*, *Salmonella typhi*, *Shigella fowleri*, and *Staphylococcus aureus*, with a minimum inhibitory concentration (MIC) ranging from 1.5 to 40 μg/mL, 3 to 6 μg/mL, 3 to 40 μg/mL, and 75 to 80 μg/mL, respectively. Additionally, the extracts showed fungicidal activity against *Fusarium oxysporum* and *Fusarium solani* with MICs of 350 to 360 μg/mL and 190 to 290 μg/mL, respectively. The authors speculated that the fungicidal activity might be attributed to the flavonoid and phenolic compounds in the extracts. In the same group, Khan et al. (66) further studied the phytochemical characteristics, antibacterial and antifungal activities of crude methanolic extracts of above-ground parts of *P. verticillatum* and their different subsequent solvent fractions. The results demonstrated that the extracts with high contents of saponins and flavonoids displayed significant antibacterial activity against a diverse range of pathogens. Consequently, the authors indicated that flavonoids in the genus *Polygonatum* possessed significant antibacterial activity. Wang et al. (67) isolated five isoforms of isoflavones from *P. odoratum* rhizomes. The compounds were found to be inhibitory against four bacterial and six phytopathogenic species. The C-6 methylated isoflavones were identified as the most active. It could be reasonably deduced that a reduction in the polarity of these isoflavones, achieved through C-methylation or O-methylation of C-8 or C-4′, might lead to an increase in their inhibitory activity (67).

### 3.3 Antioxidant activity

Excessive oxidation in the body can result in a large number of free radicals. When the clearance limit of antioxidant enzymes is exceeded, it can lead to many adverse effects, including mitochondrial damage, tissue damage, slowed metabolic processes, accelerated ageing, and other complications. The potential use of antioxidants to protect against a range of physiological and pathological processes induced by free radical reactions has attracted considerable interest. Many studies have showed that flavonoids from the genus *Polygonatum* exhibited antioxidant activity (Table 1). Flavonoids are a large group of phenolic hydroxyl groups, which generally exert antioxidant capacity through various mechanisms, such as scavenging free radicals, reducing ROS content, increasing gene expression of antioxidant enzymes, regulating Nrf2 and MAPK signalling pathways, among others (22, 23, 25, 26). Collectively, these actions contribute to the reduction of the damage caused by oxidative stress to the human body. Homoisoflavonoids, one of main flavonoids in the genus *Polygonatum*, have been demonstrated to exhibit a range of beneficial effects, such as anti-diabetic, antioxidant, and anti-inflammatory properties. Furthermore, it is well established that the beneficial effects of the active constituents in the treatment of diabetes and inflammation are related to their antioxidant activity. The antioxidant activity of flavonoids is dependent on their structures, contents, and interactions with other compounds. Furthermore, the absorption and bioavailability of flavonoids are affected by food processing, gut microbiota, and interindividual variations (22, 23). Wang et al. (68) identified the crude extracts of flavonoids from *Polygonatum* rhizomes as the source of the strongest antioxidant activity. The extracts were purified to yield two C-methylated homoisoflavanones. These two compounds demonstrated remarkable antioxidant activity and reducing capacity against DPPH radicals. The authors indicated that the C-methylated isoform of homoisoflavanones might possess the capacity to mitigate oxidative stress. Zhou et al. (69) isolated eleven homoisoflavonoids from *P. odoratum*. The authors performed DPPH scavenging experiments to evaluate the free radical scavenging ability of these homoisoflavonoids. The findings revealed that all of the homoisoflavonoids exhibited strong free radical scavenging capacity. Further interpretation of the various spectral data demonstrated that the number of OH groups present on the *β*-ring had a significant effect on their scavenging ability. Compounds with a dihydroxylated *β*-ring showed enhanced DPPH radical scavenging activity compared to the positive control ascorbic acid. In contrast, methylation at position 40 and dehydroxylation at position 20 resulted in a reduction in the scavenging activity of the compounds for DPPH radicals. Compound without OH group on the β-ring, exhibited the lowest antioxidant activity (69).

**Table 1:** Antioxidant activity of flavonoids from the genus *Polygonatum.*

Sources	Compounds	Assay methods	Results	References
*P. cyrtonema*	Water extracts	ABTS, DPPH, FRAP	ABTS IC_50_ 1800 (μg/mL)	(110)
			DPPH IC_50_ 500 (μg/mL)	
*P. sibiricum*	Ethyl acetate fraction	ABTS, DPPH, FRAP	ABTS IC_50_ 90.47 (μg/mL)	(73)
			DPPH IC_50_ 8.96 (μg/mL)	
			FRAP value 2.59 mM Fe^2+^/g	
*P. kingianum*	The flavonoids	DPPH, ABTS	DPPH IC_50_ 2.11 (mg/mL)	(111)
			ABTS IC_50_ 1.63 (mg/mL)	
*P. cyrtonema*	The flavonoids	DPPH, OH	At a concentration of 120 μg/mL, the scavenging rates of DPPH and hydroxyl radicals were comparable to that of Vc.	(112)
*P. kingianum*	The flavonoids	DPPH, OH	At a concentration of 0.50 mg/mL, the scavenging rates of DPPH and hydroxyl radicals were comparable to that of Vc.	(113)
*P. sibiricum*	Total flavonoids	DPPH	At a concentration of 0.50 mg/mL, the scavenging rate of DPPH reached to 91%.	(114)
*P. sibiricum*	Total flavonoids	DPPH, ABTS	When the concentration was from 2.0 to 3.0 mg/mL, the scavenging rate of ABTS was comparable to that of Vc.	(115)
*P. cyrtonema*	Total flavonoids	DPPH	The highest scavenging rate of DPPH reached to 89.38%.	(116)
*Alcoholic polygonatum*	Total flavonoids	DPPH, ABTS, OH	ABTS IC_50_ 31.57 (μg/mL)	(117)
			DPPH IC_50_ 19.43 (μg/mL)	
			OH IC_50_ 4.46 (μg/mL)	
*P. sibiricum*	Total flavonoids	DPPH, ABTS, OH	ABTS IC_50_ 23.75 (μg/mL)	(118)
			DPPH IC_50_ 11.20 (μg/mL)	
			OH IC_50_ 57.88 (μg/mL)	
*P. sibiricum*	Total flavonoids	DPPH, ABTS, FRAP	ABTS IC_50_ 11.47 (μg/mL)	(119)
			DPPH IC_50_ 27.55 (μg/mL)	
			FRAP IC_50_ 32.26 (μg/mL)	

Nie et al. (70) studied the effects of steaming process on homoisoflavonoids constituents and antioxidant activity of *P. cyrtonema* rhizomes. The results revealed that the steaming process altered the rhizomes’ structure, resulting in the release of higher levels of free flavonoids. In investigating the correlation between homoisoflavonoids and bioactivity of steamed *P. cyrtonema*, the researchers found that a lower the IC_50_ value indicated a greater capacity to inhibit free radicals and enzyme activities. As the extent of steaming increased, a consistent upward tendency was demonstrated in the nine homoisoflavonoids, and it was found that the higher the extent of steaming resulted in stronger antioxidant activity in the rhizomes. Consequently, steaming was an effective method of releasing homoisoflavonoids from the binding fibers of *P. cyrtonema* rhizomes, thereby enhancing their antioxidant activity. Teng et al. (71) reported increased total flavonoids and total phenolic contents and enhanced antioxidant and hypoglycaemic activities in the alcoholic extract of *P. cyrtonema*. These findings further substantiated the hypothesis that the enhanced antioxidant ability and *α*-glucosidase inhibitory activity of *P. cyrtonema* were correlated with the increased content of flavonoids and polyphenols during the processing.

At present, the antioxidant activity of flavonoids from the genus *Polygonatum* has been evaluated *in vitro* and *in vivo*. *In vitro* evaluation items are typically employed, including DPPH scavenging rate, ferric reducing antioxidant powder (FRAP), OH radical inhibition rate. *In vivo* assay, the evaluation has generally been carried out on rats. Zhao et al. (72) found that all four parts of *P. cyrtonema* exhibited antioxidant activity when assessed using three antioxidant assays: DPPH scavenging rate, OH radical inhibition rate, and FRAP. The antioxidant activity of each part of *P. cyrtonema* was found to be in accordance with the flavonoids content of each part of *P. cyrtonema*, with the order of the magnitude of antioxidant activity being as follows: leaf>flower>stem>root. It could thus be concluded that antioxidant activity of *P. cyrtonema* was mainly dependent on total flavonoids content of *P. cyrtonema*. Similarly, Li et al. (73) evaluated antioxidant activity of *P. sibiricum* using three kinds of assays: ABTS, DPPH, and FRAP. The findings indicated that ethyl acetate (EA) fraction showed the highest radical scavenging capacity for both DPPH and ABTS, as well as the highest FRAP. Additionally, the EA fraction demonstrated a higher content of total flavonoids, which further substantiated the hypothesis that *Polygonatum*’s antioxidant activity was related to its total flavonoids. Chen et al. (74) isolated the flavonoid isoquercetin from *P. sibiricum* and determined its antioxidant activity. The compound demonstrated a potent capacity to scavenge DPPH radicals and ABTS, and its antioxidant capacity exceeded that of the positive control, butylhydroxytoluene, in the FRAP assay. Isoquercetin demonstrated a higher anti-*α*-glucosidase effect than the positive control acarbose, a higher anti-acetylcholinesterase activity than the positive control chlorogenic acid, a more potent inhibitory effect on NO than the positive control quercetin, and a marked inhibition of the production of TNF-α, iNOS, and IL-6 in a concentration-dependent manner, thereby exhibiting effective anti-inflammatory activity.

Using an exhaustive treadmill test, Horng et al. (75) determined the total antioxidant activity, malondialdehyde levels, and superoxide dismutase (SOD) activities in the extracts of *P. altelobatum Hayata* (EPA)-administered exercising mice. Exercise was showed to increase lipid peroxidation and decrease antioxidant activity (76). Conversely, EPA was shown to up-regulate antioxidant enzyme activity and prevent the damage of lipid peroxidation, protecting the body from exercise-induced oxidative stress. The authors demonstrated that the antioxidant activity of EPA was found to be associated with the total flavonoids, total polyphenols, and polysaccharide compounds in its composition. However, the antioxidant activity of flavonoids was not further analysed in this study. In the normoxic hypoxia constraint model, it was observed that the longer the survival time, the stronger the resistance to hypoxia. Li et al. (77) reported that the aqueous extracts of *P. kingianum* significantly prolonged the hypoxic survival time in rats through a series of normoxic hypoxic restraint experiments. The positive control in these experiments was Rhodiola capsules, and the effect of the dose of 8 g/kg/d dose was found to be similar to that of the Rhodiola capsules group. Furthermore, the aqueous extracts of *P. kingianum* were observed to exert their anti-hypoxic effect primarily through the components of (6aR,11aR)-10-hydroxy-3,9-dimethoxyterpeneane, neoliquiritin, liquirigenin, and the targets of AKT, HIF-1α, VEGFA, and IL-6, as revealed by the network pharmacological analysis. Cardiomyocyte viability was significantly reduced under hypoxic conditions. In contrast, treatment with 20 μM and 80 μM liquiritigenin (flavonoids) led to a marked improvement in cardiomyocyte viability, accompanied by a significant improvement in SOD activity and a marked decrease in serum lactate dehydrogenase (LDH) activity. Oxygen radical damage results in direct peroxidative damage to cellular components, and increased SOD activity protects cells from oxygen radical damage (78). LDH, as a glycolytic enzyme, plays a crucial role in the glycolysis and gluconeogenesis pathways in organisms. However, elevated levels of LDH activity can result in increased levels of lactic acid accumulation in tissues, as well as more pronounced energy metabolism disorders *in vivo* (79). The aforementioned results demonstrated that liquiritigenin, present in the aqueous extract of *P. kingianum*, was able to exert an anti-hypoxic effect by inhibiting oxidative stress damage. This evidence substantiated the assertion that liquiritigenin was a major active component in *P. kingianum* with an anti-hypoxic property.

### 3.4 Anti-inflammatory activity

Inflammation represents a series of intricate physiological changes produced by the organism in response to external stimuli. It has been frequently observed that inflammatory responses are often accompanied by a number of associated phenomena, including pain, increased vascular permeability, protein denaturation, and alterations in cell membranes (2, 80). It is generally accepted that flavonoids are capable of exhibiting anti-inflammatory activity through a variety of mechanisms. These include the reduction of pro-inflammatory factors secretion (e.g., interleukin-1β, and interliukin-6) and gene expression of enzymes (e.g., inducible nitric oxide synthase, and cyclooxygenase-2). Additionally, flavonoids have been observed to regulate signalling pathways (e.g., MAPK, and NF-κB), thereby reducing the damage caused by diseases associated with inflammation (21–23, 25). The genus *Polygonatum* exerts the anti-inflammatory effect via a multi-component, multi-target, and multi-pathway mechanism, and flavonoids from the genus *Polygonatum*, have been demonstrated to have anti-inflammatory activity (56, 81, 82). Singh and Patra (83) found that the concentration of total flavonoids in *P. verticillatum* extracts exhibited a remarkable positive correlation with the inhibition of protein denaturation. The authors indicated that the total flavonoids present in the extracts possessed anti-inflammatory potential. The anti-inflammatory activity of flavonoids derived from *Polygonti* rhizome was found to be significantly superior to that of other species, particularly homoisoflavones. In addition, Gao (84) evaluated the anti-inflammatory activity of the ethanolic extracts of *P. cyrtonema* using lipopolysaccharide-induced RAW264.7 cells. The findings indicated that flavonoids exhibited the most pronounced anti-inflammatory activity, with flavonoid H demonstrating the strongest inhibitory effects on NO production in a dose-dependent manner.

Many enzymes have been showed to play a significant role in the pathogenesis of various human diseases. Lipoxygenases (LOXs) are dioxygenases that facilitate the conversion of polyunsaturated fatty acids (e.g., linoleic acid, arachidonic acid) to peroxides. LOX enzymes are expressed in immune, epithelial and tumor cells and exhibit various physiological functions, such as the process of inflammation, skin diseases, and tumor formation (85). Khan et al. (86) found that the methanolic extracts of above-ground parts of *P. verticillatum* and its subsequent solvent fractions demonstrated significant enzyme inhibition of LOX. The authors indicated the constituents, including saponins, alkaloids, flavonoids, and other constituents, had the potential to inhibit the associated inflammatory responses induced by LOXs. Human leukocyte elastase represents a major class of *in vivo* human proteases that act on a range of substrates, including elastin, collagen, and proteoglycans, within various connective tissues, including the lung, kidney, and liver. Elastase plays a pivotal role in tumorigenesis and metastasis due to its ability to destroy collagen fibers and basement membranes, thereby making it a novel antitumor drug target (87). Li et al. (88) examined the elastase inhibitory potential of three homoisoflavonoids compounds derived from *P. odoratum* extract in human leukocytes. The findings indicated that all three compounds exhibited a concentration-dependent relationship for human leukocyte elastase inhibition, suggesting their efficacy as anti-inflammatory agents.

### 3.5 Anticancer activity

It has been demonstrated that the formation of free radicals, including reactive oxygen species (ROS) and reactive nitrogen species, represents a significant contributing factor to be development of cancer. These free radicals are generally recognized as mutagenic and carcinogenic agents (83, 89). Flavonoids have been demonstrated to possess anticancer properties, due to their ability to effectively mitigate oxidative stress and the generation of excessive free radicals. Accumulating evidence has confirmed that homoisoflavones possess a range of biological properties, including cytotoxicity, which can result in the apoptosis of cancer cells (90, 91). Rafi and Vastano (92) isolated two homoisoflavonoid compounds from *P. odoratum*, which were observed to induce the phosphorylation of B-cell lymphoma 2 (Bcl-2, an anti-apoptotic protein that is highly expressed in numerous cancers) and block the G2/M cell-cycle reaction, thereby promoting the apoptosis of cancer cells and exerting the anticancer effect. In one other study, Li et al. (88) isolated three homoisoflavonoid compounds from *P. odoratum*, which also demonstrated inhibitory effects on cell growth in K562, A549 and HCT-15 cells. Consequently, they exhibited strong anticancer activity. Ning et al. (93) reported that homoisoflavanone-1, which was obtained from *P. odoratum*, significantly inhibited the growth of tumor cell and induced the apoptosis of A549 non-small cell lung cancer cells in a dose-dependent manner. The anticancer mechanism of this compound might be due to mitochondrial-mediated apoptosis and the p38 mitogen-activated protein kinase. Recently, Guo et al. (94) also reported that the flavonoids extracts of *P. sibiricum* exhibited dose- and time-dependent anti-A549 cell proliferation *in vitro* by CCK-8 assay.

Abnormal cell proliferation cycle regulation and disruption of the apoptosis balance may result in malignant tumors (95–98). The Bcl-2 family and its related proteins play a pivotal role in apoptosis (92). Representative proteins that regulate this process include the anti-apoptotic protein Bcl-2 and the pro-apoptotic protein Bax. The ratio of Bcl-2 to Bax determines the activity and death of cancer cells. Furthermore, the activation and expression of the apoptosis protease, caspase, has also been demonstrated to induce the apoptosis of cancer cells. Additionally, the formation of new blood vessels is a prerequisite for promoting tumor growth and metastasis. In the absence of new blood vessel formation, tumor cells will undergo necrosis due to a lack of nutrients, oxygen, and growth factors. Flavonoids have been shown to play a significant role in anticancer activities by several mechanisms, including the inhibition of cancer cell proliferation, the induction of apoptosis, tumor growth, and new angiogenesis. The diverse range of anticancer mechanisms makes flavonoids promising candidates for future development as anticancer drugs (96, 97). The anticancer molecular mechanism of rhizoma *Polygonatum* has been explored. Yang et al. (99) investigated the anti-non-small cell lung cancer (NSCLC) activity of rhizoma *Polygonatum* using network pharmacology and molecular docking. The results showed that 8 components in rhizoma *Polygonatum* exerted anti-NSCLC effects through 43 targets; these mainly included cancer, apoptosis, hypoxia inducible factor-1, tumor necrosis factor and other signalling pathways; 3′-methoxydaidzein, baicalein, liquiritigenin, and 5,4′ -dihydroxyflavone played major roles corresponding to 7 targets; the IC_50_ of 3′-methoxydaidzein, baicalein, and liquiritigenin acting on A549 cells for 48 h were 6.88 × 10, 7.99, and 4.34 × 10 μg/mL respectively; high-dose baicalein could induce apoptosis of A549 cells in a content-dependent manner. The authors indicated that flavonoids in rhizoma *Polygonatum* could suppress proliferation and induce apoptosis to play the role of anti-NSCLC.

### 3.6 Anti-fatigue activity

Excessive exercise can lead to oxidative stress, which can cause muscle damage and fatigue, as well as a decline in physical performance. Researchers are currently investigating the potential of a natural product to enhance exercise performance while simultaneously reducing fatigue (4). Some investigations have demonstrated that flavonoids may have an anti-fatigue effect. The most straightforward way of verifying whether the mice are fatigued is to observe their exercise endurance. In the work of Horng et al. (75), it was observed that the 20% ethanol extract of *P. altelobatum Hayata* (EPA) exhibited some anti-fatigue activity in exercising rats. In the exhaustive treadmill test, the endurance of rats supplemented with EPA was significantly superior to that of rats without added EPA. In the trend analysis, the endurance running time of rats supplemented with EPA exhibited a dose-dependent relationship, with running time approximately 1.43 to 1.62 times longer than that of rats without added EPA. Therefore, EPA significantly prolonged the endurance to fatigue time in rats, and improved exercise tolerance. The total flavonoids, total polyphenols and polysaccharides in EPA might be associated with its anti-fatigue activity, however, the exact mechanism was still under investigation. Recently, Du et al. (100) observed that in the anti-fatigue experiments on mice, the experimental group that received flavonoids exhibited improved weight-bearing swimming time, hypoxia time, LDH activity, liver glycogen content, and myoglycogen content in comparison to the blank group. The similar findings were presented in the work of Yang et al. (101). Flavonoids from rhizoma *Polygonatum* were found to have an anti-fatigue activity in mice and acted as an efficient free radical scavengers, enhancing antioxidant capacity and alleviating tissue lipid peroxidation induced by excessive exercise (101). These findings suggested that flavonoids from the genus *Polygonatum* possessed anti-fatigue activity in response to exercise.

Hypoxia is a fundamental factor in the development of fatigue and the ageing process, and is associated with the onset and progression of numerous pathological conditions. The active ingredients, such as gracillin (saponins) and liquiritegenin (flavonoids), from *P. kingianum* exhibited anti-hypoxia activity. The network pharmacology results indicated that *P. kingianum* exerted its anti-hypoxia effect through a multi-component and multi-target mechanism. Additionally, it was observed that the main active constituents of *P. kingianum*, namely gracillin and liquiritigenin, played a crucial role in the anti-hypoxia activity. The anti-hypoxia activity of these compounds might be related to the scavenging rate of excess free radicals, the maintenance of antioxidant enzyme activities, and the inhibition of oxidative stress due to lipid peroxidation (5).

Flavonoids have also been demonstrated to improve cognitive function in the brain, thereby delaying the onset of ageing. Wang et al. (102) employed network pharmacology to study the potential mechanism of rhizoma *Polygonatum* in treating cognitive impairment. Their findings revealed that four key compounds present in rhizoma *Polygonatum*, namely 5,4′-dihydroxyflavone, baicalein, diosgenin and *β*-sitosterol, had the ability to interact with a greater number of targets. Similarly, Ke et al. (103) revealed the molecular mechanism of anti-ageing of rhizoma *Polygonatum* via network pharmacology and molecular docking. The active constituents in rhizoma *Polygonatum*, such as baicalein, 4′, 5-dihydroxyflavone, liquiritin and other active constituents could exhibit anti-ageing activity through multi-target and multi-pathway.

### 3.7 Lipid regulation ability

Dyslipidaemia is widely recognized as one of major contributors to the development of cardiovascular disease, particularly in the pathogenesis of atherosclerosis. Flavonoids from the genus *Polygonatum* have been indicated to show cardioprotective properties through a number of mechanisms, such as anti-oxidant, anti-inflammatory, and lipid-modulating properties. Additionally, the genus *Polygonatum* has been showed to possess a diverse spectrum of therapeutic effects on pharmacological cardiotoxicity. Xanthine oxidase (XOD) is a key flavoprotein enzyme involved in the xanthine-catalyzed generation of uric acid and superoxide anion radicals. Excessive XOD activity has been linked to the development of cellular oxidative stress, which, in turn, increases ROS levels, potentially resulting in cardiovascular tissues damage (104). Li et al. (73) identified N-cis-p-coumaroyltyramine as a potential ligand for XOD in ethyl acetate extracts of *P. sibiricum*. Molecular docking findings revealed that the compound in the extracts interacted with XOD through hydrogen bonding and other different interaction forces. The authors found that the extracts had the highest total flavonoid content, indicating that flavonoids in *Polygonatum* might have potential anticardiovascular properties. It was suggested that further investigation into its action mechanisms of the flavonoids could establish the genus *Polygonatum* as functional foods or natural medicines for cardiovascular disease and other related diseases.

Presently, pharmaceuticals for weight loss are costly and have numerous effects. Natural products have significant potential in the treatment of obesity and are expected to be a safe and effective alternative to pharmaceuticals for weight loss. Some investigations have demonstrated that the extract of *P. odoratum* has a beneficial effect on the regulation of blood lipids and weight loss. In the phenomenon of diet-induced metabolic disorders in rats, 70% ethanol extracts of *P. odoratum* were observed to reduce total cholesterol and fasting blood glucose levels, while also demonstrating efficacy in alleviating obesity caused by a high-fat diet. In addition, pancreatic lipase plays a key role in regulating the body’s fat metabolism. Inhibition of the enzyme activity would consequently lead to the decomposition of fat, thereby resulting in a reduction in body weight (105). Wang et al. (106) isolated 11 kinds of flavonoids from the above-ground parts of *Polygonatum*, including isomonycin, isoorientin, orientin, orientin 2″-O-xyloside etc., which were dominated by carbonyl glycoside flavonoids. These compounds demonstrated certain inhibitory activity against pancreatic lipase, with the inhibition rate increasing in proportion to the content of the compounds. At a concentration of 5 mM, the inhibition rate reached over 75%, with isorhynchophyllin exhibiting an inhibition rate of 83. 67%.

## 4 Conclusion and future perspectives

Flavonoids represent an important functional component of the genus *Polygonatum*, and have been have attracted continuous investigations over recent decades. The exploration of flavonoids in the genus *Polygonatum* has been initiated, and the prospective nutritional values of flavonoids have been confirmed. This work presented a review of the biosynthesis and biological activity of flavonoids. Notwithstanding the considerable advancements achieved in the research of flavonoids in the genus *Polygonatum*, a number of significant questions remain unanswered.

Firstly, despite the rapid advancements made in sequencing technology, only a limited number of potential key genes encoding key enzymes involved in the biosynthesis pathway of flavonoids in the genus *Polygonatum* have been predicted. In addition, the regulation mechanism of flavonoid biosynthesis in the genus *Polygonatum* is intricate and has not yet been fully elucidated. It is therefore imperative to gain further insight into the biosynthesis of flavonoids. Omics technologies (e.g., transcriptome, metabolome, and proteome) have revealed the dynamic changes of flavonoid compounds and the corresponding functional genes and transcription factors. At present, given the rapid development of omics research, it provides a valuable opportunity to investigate the biosynthesis metabolisms of flavonoids in the genus *Polygonatum*. However, as indicated by various studies, single omics analyses are inadequate in addressing the metabolic regulation problem. Therefore, an integrated multipleomics analysis is recommended for studying changes in gene and protein expression levels and metabolite abundance in flavonoids biosynthesis pathways. It is evident that flavonoids play important protective roles against biotic and abiotic stresses. However, our knowledge of flavonoids under various stresses is still incomplete and requires further research.

Secondly, current investigations have demonstrated that crude flavonoids are mainly used in animal experiments, but have failed to elucidate the bioactive centers of flavonoids with sufficient clarity. Therefore, future studies should focus on the investigation of the monomeric constituents and their associated pharmacological mechanisms, which would establish a solid foundation for the extensive utilization of flavonoids derived from the genus *Polygonatum*.

Thirdly, despite the numerous advantageous effects of flavonoids, their low bioavailability and solubility limit their widespread use. The future solution to this issue is the extensive use and development of nanotechnology. The applications of nanotechnology for the targeted delivery of flavonoids to improve their bioavailability are well-established. Numerous nano delivery systems including liposome, nanoparticles, solid lipid nanoparticles, and nanostructured lipid carriers, have been developed for this purpose (107–109). However, until now, the limited investigation of nano flavonoids delivery systems *in vivo*, has been carried out. In the near future, clinical trials should be attempted to study the effectiveness of these systems in delivering flavonoids from the genus *Polygonatum*.

Fourthly, flavonoids are typically administered independently. Subsequent research may explore the potential of combining of flavonoids with many other active constituents or clinical agents to make treatments more effective or to mitigate the adverse effects of drugs. The investigation of flavonoids is currently limited to the cellular and animal levels. Given the favourable safety and effective profile, there is a need to focus more on preclinical experiments.

Finally, a substantial number of reports have been published investigating the activities of flavonoids and their potential for commercial use. Nevertheless, there is a lack of extensive research on their use as functional food constituents, and there is limited data on their activities after incorporation into foods. It is therefore recommended that further investigation be carried out into the applications of flavonoids.

In conclusion, previous studies have provided a comprehensive and solid foundation for the further research of flavonoids. However, further research is highly required to facilitate the advancement and application of flavonoids. It should encompass a deeper understanding of their biosynthetic pathway, activity mechanisms, and potential clinical applications.
